# Generation of Knockout Rats with X-Linked Severe Combined Immunodeficiency (X-SCID) Using Zinc-Finger Nucleases

**DOI:** 10.1371/journal.pone.0008870

**Published:** 2010-01-25

**Authors:** Tomoji Mashimo, Akiko Takizawa, Birger Voigt, Kazuto Yoshimi, Hiroshi Hiai, Takashi Kuramoto, Tadao Serikawa

**Affiliations:** 1 Institute of Laboratory Animals, Graduate School of Medicine, Kyoto University, Kyoto, Japan; 2 Shiga Medical Center for Adult Disease, Moriyama, Japan; University Medical Center Groningen, Netherlands

## Abstract

**Background:**

Although the rat is extensively used as a laboratory model, the inability to utilize germ line-competent rat embryonic stem (ES) cells has been a major drawback for studies that aim to elucidate gene functions. Recently, zinc-finger nucleases (ZFNs) were successfully used to create genome-specific double-stranded breaks and thereby induce targeted gene mutations in a wide variety of organisms including plants, drosophila, zebrafish, etc.

**Methodology/Principal Findings:**

We report here on ZFN-induced gene targeting of the rat interleukin 2 receptor gamma (*Il2rg*) locus, where orthologous human and mouse mutations cause X-linked severe combined immune deficiency (X-SCID). Co-injection of mRNAs encoding custom-designed ZFNs into the pronucleus of fertilized oocytes yielded genetically modified offspring at rates greater than 20%, which possessed a wide variety of deletion/insertion mutations. ZFN-modified founders faithfully transmitted their genetic changes to the next generation along with the severe combined immune deficiency phenotype.

**Conclusions and Significance:**

The efficient and rapid generation of gene knockout rats shows that using ZFN technology is a new strategy for creating gene-targeted rat models of human diseases. In addition, the X-SCID rats that were established in this study will be valuable *in vivo* tools for evaluating drug treatment or gene therapy as well as model systems for examining the treatment of xenotransplanted malignancies.

## Introduction

Although several strategies are available for producing a wide variety of genomic alterations in the mouse, the same cannot be said of the rat. Rat ES cells [1], [2] and induced pluripotent stem cells (iPS) [3], [4] are available, but the culture conditions for these cells and the methodology for inducing homologous recombination are imperfect [5]. Rat spermatogonial stem cells (SSC) have also been isolated and cultivated *in vitro* but their yield proved unsatisfactory in terms of their ability to undergo homologous recombination [6], [7]. Besides these methods which are based on the *in vitro* genetic engineering of pluripotent stem cells, transposon-mediated mutagenesis [8] and N-ethyl-N-nitrosourea (ENU) mutagenesis [9], [10] have been used with some success for producing mutations in the rat genome. We recently reported on a high-throughput gene-driven strategy which uses the mutagen ENU and the Mu-transposition reaction (MuT-POWER) to rapidly detect induced mutations. This was in addition to our investigation of intracytoplasmic sperm injection (ICSI) for recovering heterozygous genotypes of interest out of a large sperm cell repository [11], [12]. However, even if a large number of mutant strains already exists or may potentially be available, targeted modification or disruption of specific DNA regions is difficult to achieve. Even in the case of our gene-driven strategy, X-linked mutations are impossible to obtain because of the breeding protocol which is used [11].

Recently, a novel gene-targeting technology which employs zinc-finger nucleases (ZFNs) has been proven to work successfully in plants, *Caenorhabditis elegans*, frogs, drosophila, zebrafish, and human ESCs and iPSCs [13], [14], [15]. ZFNs are chimeric proteins that consist of a specific DNA-binding domain which is made of tandem zinc finger-binding motifs that are fused to a non-specific cleavage domain of the restriction endonuclease *Fok*I. ZFNs can create site-specific double-stranded breaks which are repaired via non-homologous end joining (NHEJ), a process that results in the arbitrary addition or deletion of base pairs. Consequently, repair by NHEJ is mutagenic and results in a knockout. Recently, it was reported that a single injection of DNA or messenger RNA that encodes specific ZFNs into one-cell transgenic rat embryos that express GFP could lead to a high frequency of animals that do not express the transgenic marker as a consequence of homologous recombination at the GFP site [16]. Here, we report on an experiment that involved using ZFN technology. The aim of the experiment was to inactivate the gene that encodes the interleukin 2 receptor gamma (*Il2rg*), which is essential for signaling by interleukins such as IL-2, IL-4, IL-7, IL-9, IL-15, and IL-21. In addition, the gene is involved in the X-linked form of severe combined immunodeficiency (X-SCID), one of the most common forms of human SCID [17], [18]. A major motivation for performing this experiment was the observation that although SCID mouse animal models are the most commonly used in research on drug development, an X-SCID immunodeficient rat model would complement mouse models through the additional advantage of being employed for testing the pharmacodynamics and toxicity of potential therapeutic compounds. Following the results of research involving *Prkdc* SCID [19], [20] and *Il2rg* X-SCID mice [21], [22], [23], *Il2rg* X-SCID rats should have a very low level of NK cell activity and thereby make xenotransplantation more successful.

## Results

### Injection of *Il2rg* ZFN-encoding mRNA into rat embryos

**Figure 1:**
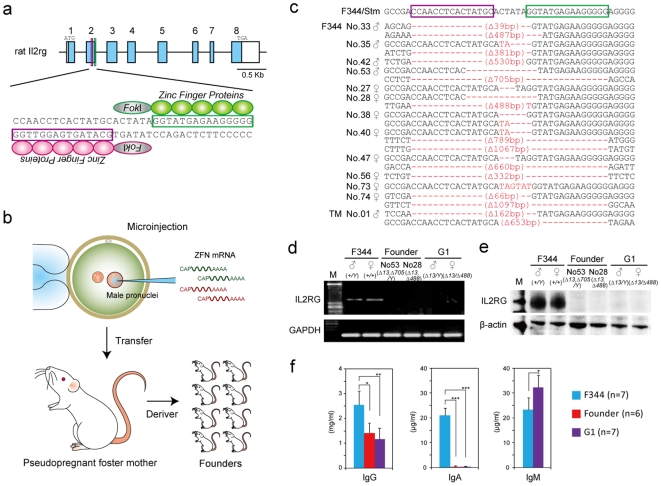
Injection of *Il2rg* ZFN-encoding mRNA into rat embryos induced targeted loss-of-function mutations. (a) Schematic representation of the rat *Il2rg* gene. Exons are represented as blue boxes. Regions used to design the ZFN templates are printed in red for the left ZFN and green for the right ZFN. The magnified views illustrate the binding sites for the ZFN pairs. Please see Figure S4 for further details. (b) Schematic representation of the method used for ZFN-targeted mutagenesis in rat embryos. (c) Sequencing assay for ZFN-induced mutations in the *Il2rg*-targeted region. Multiple deletions or insertions depicted using red dashes or letters, respectively, are aligned along the wild-type sequences shown on the top line. (d) RT-PCR analysis of IL2RG mRNA expression in the spleen of control F344, founder (G0), and G1 rats. GAPDH expression was used as an internal control. (e) Western blotting for IL2RG protein in the spleen of control F344, founder (G0), and G1 rats. β-actin was used as a loading control. (f) ELISA for serum IgG, IgA, and IgM levels in control F344, founder (G0), and G1 rats. *P<0.01, **P<0.001, and ***P<0.0001, indicated for each group in comparison with control F344 for independent sample Student t-tests.

Of 443 ZFN-injected embryos, 230 (51.9%) were transferred into the oviducts of pseudopregnant female rats, and 54 (24.3%) of these embryos were successfully carried to term as shown in Figure 1a, b and Table 1. Sequence analysis of the ZFN target site of these 54 founder animals revealed that 5 males and 8 females (24.1%) carried a variety of mutations including from 3 to1,097 bp deletions and a 1 bp insertion in the region which overlapped the ZFN target site as seen in Figure 1c and Figure S1. Four out of five of the males carried different biallelic mutations at the *Il2rg* locus despite them only having one X chromosome. This suggests that mosaicism was induced by the ZFN treatment, a situation which is frequently observed in the DNA of transgenic founders. Three of the affected females had a monoallelic homozygous mutation, four had heterologous or mosaicism biallelic mutations, and the remainder had three different mosaic mutations. The normal F344-allele was not found in the affected founder animals. Most of these mutations were expressed as frameshifts or splicing errors and resulted in no or very little IL2RG mRNA being expressed as shown in Figure 1d probably due to nonsense-mediated decay. Western blotting with antibodies against the C-terminal domain of IL2RG did not reveal any protein in the founder animals as seen in Figure 1e.

**Table 1:** Injection of ZFN-encoding mRNA into fertilized oocytes.

Strain	Oocyte state	Injected oocytes	Transferred oocytes (%)	Born (%)	Mutants (%)
F344/Stm	Fresh	234	32 (-)	♂2,♀5 (21.9)	♀2 (28.6)
	Cryopreserved^a^		57 (-)	♂8,♀10 (31.6)	♀2 (11.1)
	Fresh	182	129 (68.3)	♂16,♀11 (20.9)	♂4,♀4 (29.6)
TM/Kyo	Fresh	27	12 (44.4)	♂1,♀1 (16.7)	♂1 (50.0)
**Total**		**443**	**230 (51.9)**	**♂27,♀27 (24.3)**	**♂5,♀8 (24.1)**

a Injected oocytes were cultured in KRB overnight and cryopreserved at the two-cell stage.

To clarify whether the ZFNs only induced mutations in the targeted region, we checked 16 sites that showed a high rate of similarity with the targeted site at the sequence level with no more than 6 to 7 bp mismatches as illustrated in Table S1. Insertions or deletions were not observed at any of these off-target sites among the 13 ZFN-modified founders. This confirms that ZFNs can be reliably and efficiently used to produce mutant alleles at loci of interest. Although we cannot exclude the possibility that the ZFNs may have cleaved unknown off-target sites, such undesired mutations can subsequently be easily excluded from the genome of the carrier animals by backcrossing to the parental strain or another background strain.

### Germ line transmission of ZFN-modified genetic changes

To assess the transmission of ZFN-modified genetic changes to the next generation, we crossed the founder animals with the background strain F344/Stm as depicted in Table S2. All 38 offspring consisting of 18 males and 20 females that were obtained from the founder females mated with the F344 males had one of the maternal mutations. This indicates that ZFN-induced mutations were faithfully transmitted through the germ line. In the offspring that were obtained from the founder males, there were two cases where only one of the paternal bialleles was transmitted or both alleles were transmitted. This suggests that mosaicism occurred not only in somatic cells but also in the germ line of the founder animals. PCR analysis of genomic DNA isolated from several types of tissues indicated that somatic mosaicism occurred in the progenitors but not in their offspring as shown in Figure S2.

**Figure 2:**
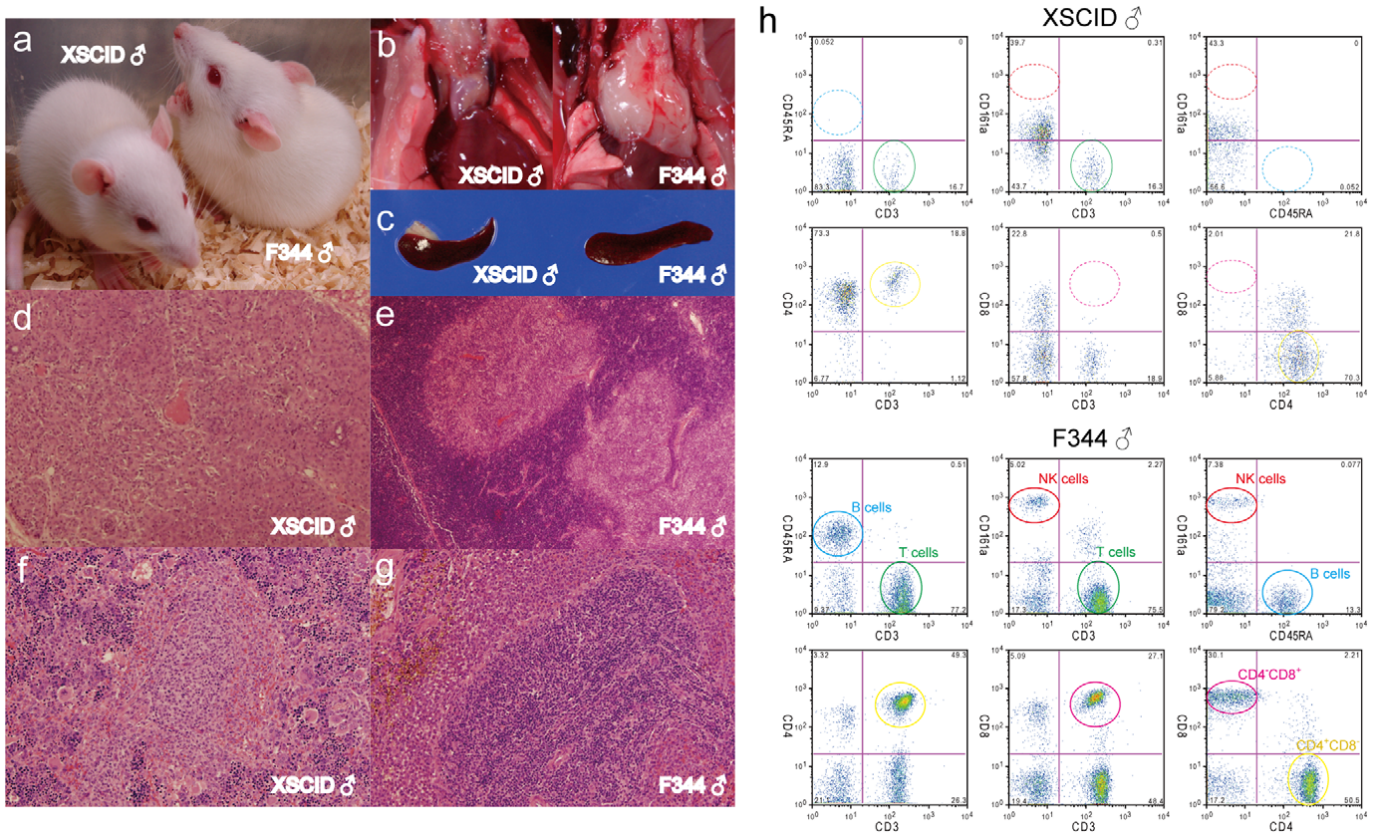
Abnormal lymphoid development in X-SCID rats. (a) Photograph of five week-old male X-SCID (*Il2rg^−^/Y*) and F344 (*+/Y*) rats. (b) Thymus of X-SCID and F344 rats. (c) Spleen of X-SCID and F344 rats. (d, e) Histological analysis of the thymus of X-SCID (X40) and F344 (X40) rats. The thymus of the X-SCID rat was severely hypoplastic and consisted of an epithelial cell sheet. (f, g) Histological analysis of the spleen of X-SCID (X100) and F344 (X100) rats. In the X-SCID spleen, the white pulp was virtually devoid of lymphocytes and the red pulp was occupied by a variety of myeloid elements. (h) Dot plots representing CD3, CD45RA and CD161a for differentiation of T-, B- and NK cell sub-populations, and CD3, CD4 and CD8 for demarcation of T-cell sub-populations in peripheral blood lymphocyte cells. The numbers shown in the quadrants are mean percentages. The circled areas indicate cell populations that are referred to in the text.

We intercrossed the G0 founders to produce hemizygous males (*Il2rg^−^/Y*) and homozygous females (*Il2rg^−^/Il2rg^−^*) for the ZFN-induced mutation listed in Table S3 to characterize the immunodeficient phenotypes of the X-SCID rats. The hemizygous males and homozygous females appeared normal at birth and developed well as shown in Figure 2a. RT-PCR and Western blot assays was performed on these G1 rats and the results showed a complete loss of expression of the *Il2rg* gene as detailed in Figures 1d, e. ELISA for serum immunoglobulin (Ig) levels revealed reduced IgG, diminished IgA, and increased IgM levels in the G1 rats as noted in Figure 1f.

### Characterization of *Il2rg-*deficient X-SCID rats

Gross and microscopic analyses at five weeks of age showed that the X-SCID rats underwent abnormal lymphoid development as depicted in Figure 2. The thymus of X-SCID rats was extremely hypoplastic as seen in Figure 2b and consisted of an epithelial rudiment without any lymphocytes as seen in Figure 2d. The spleen was moderately decreased in size as noted in Figure 2c, and the white pulp was severely hypoplastic and the red pulp contained myeloid cells as shown in Figure 2f. Peripheral lymph nodes and Peyer's patches were not identified by necropscopy. In the peripheral blood (PB) profiles, the numbers of white blood cells (WBCs) was reduced compared to those of control rats as detailed in Table S4. Differential counts of WBCs showed a dramatic decrease in leukocytes in the X-SCID rats (Table S5). Flow cytometry analysis of cell populations isolated from PB, bone marrow (BM), and the spleen also revealed a dramatic decrease in the number of the lymphocytes as seen in Figure 2h and Figure S3. The number of CD4^−^CD8^+^ T-cells was markedly diminished and the number of CD4^+^CD8^−^ T-cells was decreased although some cells were present in PB, BM and the spleen. The numbers of CD3^−^CD45RA^+^ B-cells and CD3^−^CD161a^+^ NK cells were markedly diminished in PB and BM, but some cells were present in the spleen. Heterozygous females exhibited normal lymphoid development and were indistinguishable from normal control females (data not shown).

### Xenotransplantation of human tumor cells

**Figure 3:**
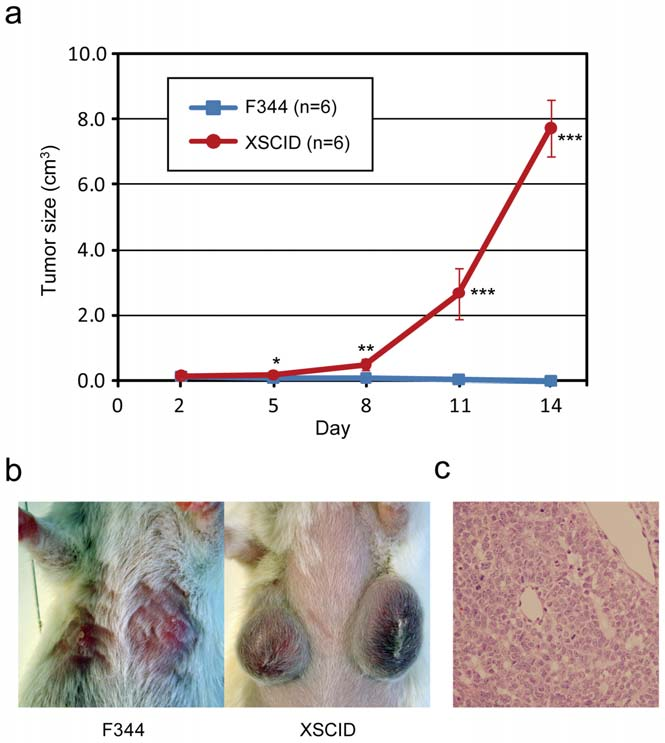
Tumor development from the xenotransplantation of human ovarian cancer cells. (a) Growth curve of tumor development after subcutaneous injection of A2780 human ovarian cancer cells in F344 and X-SCID rats. *P<0.01, **P<0.001, and ***P<0.0001, indicated in comparison with control F344. (b) The tumors became large and grew quickly about 11 days after injection in X-SCID rats but not in F344 rats. (c) Histology of the xenotransplanted tumors that formed in X-SCID rats (X400). No lymphocytic infiltration was detected in the tumors.

These immunodeficient phenotypes of the X-SCID rats were very similar to those of the previously reported X-SCID mice and were characterized by a nearly complete lack of T-cells, B-cells and NK cells [21], [22], [23]. Since X-SCID mice cannot reject transplanted tissues from other species including humans, we tested *Il2rg*-deficient rats as a host for xenotransplantation of human ovarian cancer tumor cells. All X-SCID rats developed tumors within 14 days after injection of the cells (6/6, 100%), while control F344 rats showed no tumor growth (0/6, 0%) as seen in Figure 3a, b. The tumors were confirmed by histological analysis as depicted in Figure 3c and by PCR with primers that were used to amplify the human MHC class II DQB2 region (data not shown). These observations illustrate the impaired immune system function of X-SCID rats and suggest that the animals may be important models for cancer and transplantation research.

## Discussion

In this study, we proved that targeted gene disruption using ZFN technology works well and provides for several advantages and possibilities when used in rats. First and foremost, knockout rats can be created in a four- to six-month time frame and with high efficiency at more than 20%. This is more favorable than the ES cell-based method for mice that usually takes 12–18 months. Given the high rate of germ line transmission, preliminary phenotypic analysis can be performed on G1 animals after intercrossing the initial G0 founders, thereby saving time and effort. Second, gene-targeting with ZFNs does not seem to be strain-dependent (unpublished data) and accordingly can be performed with any inbred strain. This is of great advantage since other techniques like ENU mutagenesis differ in their efficiency when used with different strains. This provides a straight forward strategy for directly employing targeted gene disruption in the existing strain, thereby bypassing tedious and time-consuming backcrossing steps that generally take two to three years to complete. Third, ZFNs can be used to induce a wide variety of allelic changes covering small or wide deletions or insertions. They may be used to produce frameshifts or small in-frame deletions such as the 3-bp deletion that we observed. Given the reports on successful ZFN-targeted gene modification or correction by homologous recombination in mammalian cell cultures [15], [24], [25], it should be feasible to archive targeted knock-in technologies that have thus been far inaccessible without rat ES cells. Finally, since ZFN technology does not rely on using species-specific embryonic stem cell lines, it should be possible to adapt it to other mammalian species such as pigs, cattle, and monkeys, where it is possible to harvest and manipulate fertilized embryos.

The X-SCID rats established in this study provide not only a valuable *in vivo* model for evaluating drug treatment or gene therapy approaches, but also a system for assaying novel anticarcinogenic effects on transplanted malignancies. There is a growing need for animal models with which to carry out *in vivo* studies using human cells, tissues or organs as chimeras such as humanized models [26], [27], [28]. X-SCID and SCID mice homozygous for *Il2rg*- and *Prkdc*- alleles with a non-obese diabetic background are a powerful tool for the xenotransplantation of human tissues or potentially human ES/iPS cells. This could lead to advances in our understanding of human hematopoiesis, immunology, cancer biology, infectious diseases, and regenerative medicine [29], [30], [31]. Humanized rats, if generated by ZFN technology, could be powerful tools for pre-clinical testing during drug development and be better models in various fields of translational research.

## Materials and Methods

### Animals

All animal care and experiments conformed to the Guidelines for Animal Experiments of Kyoto University, and were approved by the Animal Research Committee of Kyoto University. F344-*Il2rgt^tm1Kyo^* X-SCID rats are deposited at the National Bio Resource Project for the Rat in Japan (www.anim.med.kyoto-u.ac.jp/nbr).

### ZFN constructs

Custom-designed ZFNs plasmids for the rat *Il2rg* gene were obtained from Sigma-Aldrich. The design, cloning, and validation of the ZFNs was performed by Sigma-Aldrich [32]. ZFN design involved using an archive of pre-validated two-finger and one-finger modules [32], [33]. The target region was scanned for positions where modules exist in the archive. This allowed the fusion of two or three such molecules to generate a five-finger protein that recognizes a 15 bp site on the top strand and the fusion of two to three different modules that recognize a 15 bp site on the bottom strand that lies 5–6 bp away. Measurements of ZFNs for gene disruption activity were performed using the Surveyor endonuclease (CEL-1) assay as described elsewhere [34]. Final candidate ZFNs were designed to recognize a site within the boundary between exon 2 and intron 2 of the *Il2rg* gene as shown in Figure S4.

### Microinjection of ZFN mRNA

To prepare ZFN mRNA, ZFN-encoding expression plasmids were linearized with *Xho*I and extracted with phenol-chloroform by the standard method. Messenger RNA was transcribed *in vitro* using a MessageMax™ T7 mRNA transcription kit (Epicentre) and polyadenylated using a A-Plus™ Poly(A) polymerase tailing kit (Epicentre). The resulting mRNA was purified using a MEGAClear™ kit (Epicentre) and finally resuspended in RNase-free water at 10 ng/µl for each ZFN. Approximately 2–3 pL of capped mRNA were injected into the male pronuclei of zygotes by the same method that was used to microinject DNA. Pronuclear stage embryos were collected from F344/Stm and TM/Kyo females six weeks of age that had been super-ovulated by injecting them with eCG (Serotropin, Asuka Pharmaceutical Co.) and hCG (Gonatropin, Asuka Pharmaceutical Co.). They were mated with males of the same respective strain. The mRNA solution was injected and embryos were cultured in KRB at 37.5°C with 5% CO_2_ and 95% humidified air to promote their recovery. The embryos that survived were transferred to the oviduct of pseudopregnant females (Crlj∶WI, 8–12wks).

### Analysis of genome editing at ZFN target sites

Genomic DNA was extracted from the tail, brain, heart, and liver using a GENEXTRACTOR TA-100 automatic DNA purification system (Takara). PCR for each was carried out in a total volume of 15 µl under the following conditions for 35 cycles: 94°C for 3 min for 1 cycle, 94°C for 30 sec, 60°C for 30 sec, and 72°C for 1 min. The final reaction mixture for each contained 100 ng of genomic DNA, 200 µM of each dNTP, 1.0 mM MgCl_2_, 0.66 µM of each primer, and 0.4 U of Taq DNA polymerase (GibcoBRL).

For editing the ZFN cleavage site in the genome at the *Il2rg* locus, three primer sets were designed to amplify small 292-bp, middle 1509-bp, and large 3158-bp fragments as shown in Figure S4. The PCR products were directly sequenced using the BigDye terminator v3.1 cycle sequencing mix and the standard protocol for an Applied Biosystems 3130 DNA Sequencer. The products were also subcloned into the pCR4-TOPO vector (Invitrogen), and plasmid DNA was prepared and sequenced on a 3130 DNA Sequencer. All new sequence data is deposited in GenBank (GU294902-GU294925).

### Off-target site analysis

Off-target sites with the highest degree of similarity were identified by searching the rat genome (RGSCv3.4) for matches with the consensus sequence of each ZFP with appropriate spacing of 5–6 bp. A list of these target sites is provided in Supplementary Table 1. PCR primers were designed to flank the off-target sites as detailed in Table S6. Reactions were performed for the founder animals and the PCR products were directly sequenced on the 3130 DNA Sequencer.

### RT-PCR and Western blotting

Total RNA was extracted using Isogen reagent (Nippon Gene) from the spleen of five week-old rats. First strand cDNA was synthesized from 5 µg of total RNA that had been treated using DNase by using the oligo(dT)12–18 primer and SuperscriptII reverse transcriptase (Invitrogen). PCR was performed with the primers for *Il2rg* described in Figure S4 and with the *Gapdh* 5′- GGCACAGTCAAGGCTGAGAATG-3′ and 5′-ATGGTGGTGAAGACGCCAGTA-3′. Western blotting was carried out using the cell lysates from the spleens of five week-old rats by the standard method. Signals were detected with antibodies against rat IL2RG (M-20, Santa Cruz Biotechnology) and β-actin (AC-40, Sigma Aldrich).

### Immunofluorescence and histological analyses

Complete necroscopy examinations were performed on five week-old *Il2rg-*deficient and wild-type male and female rats. Peripheral blood specimens were collected from the caudal vena cava. Serum immunoglobulin (Ig) levels were measured by enzyme-linked immunosorbent assay (ELISA) using Rat IgG, IgA and IgM ELISA Quantitation kits (Bethyl Laboratories). Blood parameters for a complete blood cell count, a WBC differential, and a reticulocyte count were measured using ADVIA 2120 flow cytometry (Block Scientific). For histopathology, tissues were fixed in Bouin's fluid and embedded in paraffin. The embedded tissues were then sectioned at 5–7 µm thickness at room temperature and stained with hematoxylin and eosin to permit evaluation by light microscopy.

Flow cytometric analyses of cell populations isolated from bone marrow, the spleen and peripheral blood were carried out using IOTest Anti-Rat CD3-FITC/CD45RA-PC7/CD161a-APC (Beckman Coulter) to differentiate T-, B- and NKcell sub-populations and IOTest Anti-Rat CD3-FITC/CD4-PC7/CD8-APC (Beckman Coulter) to enumerate T-cell sub-populations. Anti-CD45 monoclonal antibodies (Beckman Coulter) were used for the intracellular staining of lymphocytes. Mouse IgM, IgG1 and IgG2a antibodies (Beckman Coulter) were used as isotype-matched controls. The cell samples were treated with FcR-blocking reagent (Miltenyi Biotec) for 10 minutes, stained with the fluorochrome-conjugated antibodies for 30 minutes, and washed three times with PBS/10% FCS. Stained cell samples were analyzed with a four-color FACS flow cytometer (FACSCalibur, Becton Dickinson) using CellQuest software (Becton Dickinson).

### Tumor cell xenotransplantation

The human ovarian cancer cell line A2780 was purchased from the European Collection of Cell Cultures (ECACC). Cells were cultured in RPMI1640 medium (GIBCO) with 10% heat-inactivated FBS (Hyclone). Subcutaneous injections of 2×10^5^ A2780 cells with Matrigel (Becton Dickinson) were performed on five week-old female rats. Tumors were measured by length (*a*) and width (*b*) in millimeters using calipers, and tumor volumes (*V*) were calculated according to the relationship *V* = *ab* ^2^/2, where *a* was the longer of the two measurements. Human-specific PCR primers were designed to amplify major histocompatibility complex class II DQ beta 2 (HLA-DQB2) at exon 4 as follows: 5′-CCTAGGGTGGTCAGACTGGA-3′ and 5′-AAAATCCCCCAAAACAAAGG-3′.

## Supporting Information

Figure S1 PCR analysis of 13 mutant founders for the zinc-finger nuclease (ZFN) target site. For the analysis of the ZFN target site at the *Il2rg* locus, three primer sets were used to amplify small (a, 292-bp), middle (b, 1509-bp), and large (c, 3158-bp) fragments for PCR. See Figure S4 for further details. PCR fragments were electrophoresed through a 1-4% agarose gel. M: DNA molecular weight marker φX174-*Hae*III digest.
(9.19 MB TIF)

Figure S2 PCR analysis of genomic DNA isolated from several tissues. Three primer sets were used to amplify small (a, 292-bp), middle (b, 1509-bp), and large (c, 3158-bp) fragments for PCR. See Figure S4 for further details. Genomic DNA (T: tail, B: brain, H: heart, L: liver) was used as a template for PCR in zinc-finger nuclease-modified founders (numbers 28, 35, 40, and 53) and G1 rats. PCR fragments were electrophoresed through a 1–4% agarose gel. M: DNA molecular weight marker φX174-*Hae*III digest or Lambda DNA-*Hind*III digest.
(6.28 MB TIF)

Figure S3 Flow cytometric analysis of bone marrow lymphocyte cells (a) and spleen lymphocyte cells (b) from five-week-old F344 and X-SCID rats. Dot plots represent CD3, CD45RA, and CD161a for discrimination of T-, B-, and NK cell subpopulations; and CD3, CD4, and CD8 for demarcation of T cell subpopulations. The numbers shown in quadrants are mean percentages. Circled areas indicate cell populations referred to in the text.
(6.66 MB TIF)

Figure S4 Zinc-finger nuclease pairs designed against the *Il2rg* locus and primer sequences used for PCR analysis for the *Il2rg* gene. Each exon is underlined. The start codon is indicated by a red box. The three primer sets (small, middle, and large) used for the PCR analysis of *Il2rg* are shown by boxes. Primers used for the RT-PCR are shown as cDNA.
(3.32 MB TIF)

Table S1 Potential zinc-finger nuclease off-target sites.
(0.14 MB DOC)

Table S2 Backcrossing of zinc-finger nuclease-modified founders to F344/Stm rats.
(0.16 MB DOC)

Table S3 Intercrossing of zinc-finger nuclease-modified founders between males and females.
(0.08 MB DOC)

Table S4 Peripheral blood profiles of *Il2rg*-deficient (X-SCID) rats.
(0.09 MB DOC)

Table S5 Differential counts of the white blood cells of *Il2rg*-deficient (X-SCID) rats.
(0.07 MB DOC)

Table S6 Primer sequences for zinc-finger nuclease off-target analysis.
(0.14 MB DOC)
