# Supplementary figures and images for: Generation of Knockout Rats with X-Linked Severe Combined Immunodeficiency (X-SCID) Using Zinc-Finger Nucleases
Source: PLoS One. 2010 Jan 25;5(1):e8870. doi: 10.1371/journal.pone.0008870 (PMC2810328; doi:10.1371/journal.pone.0008870)

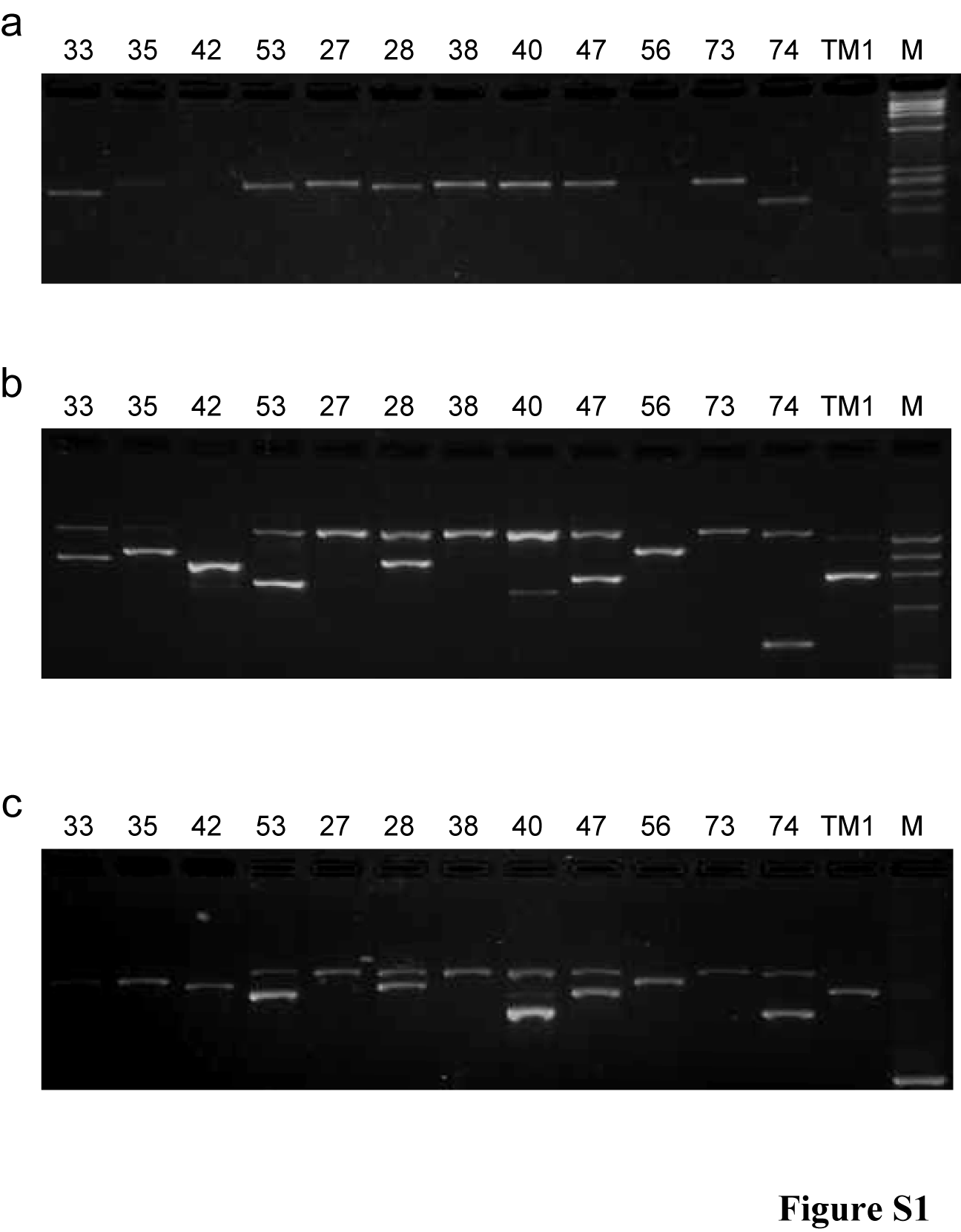

Supplement: Figure S1 — PCR analysis of 13 mutant founders for the zinc-finger nuclease (ZFN) target site. For the analysis of the ZFN target site at the Il2rg locus, three primer sets were used to amplify small (a, 292-bp), middle (b, 1509-bp), and large (c, 3158-bp) fragments for PCR. See Figure S4 for further details. PCR fragments were electrophoresed through a 1-4% agarose gel. M: DNA molecular weight marker φX174-HaeIII digest. (9.19 MB TIF) [file pone.0008870.s001.tif]

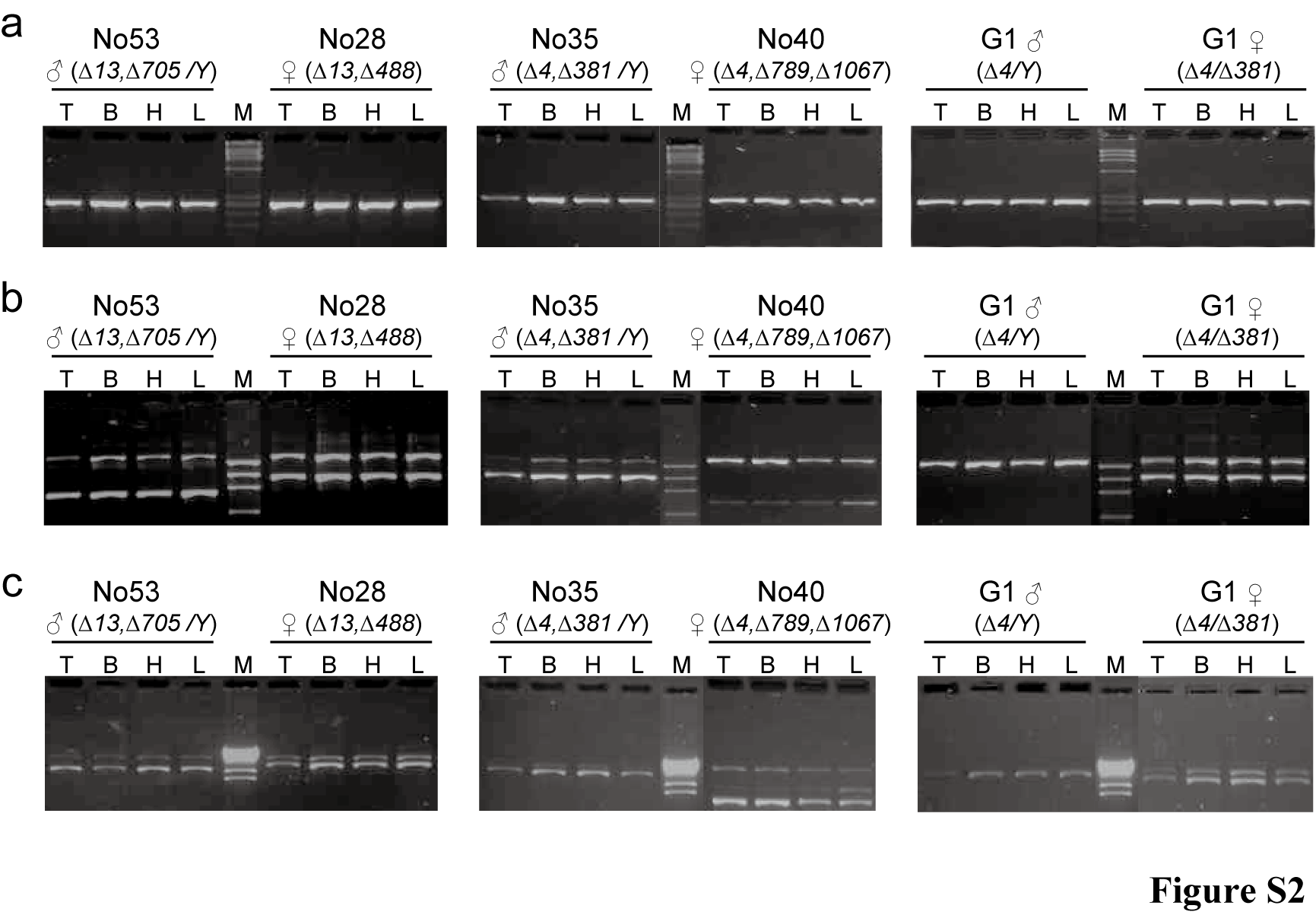

Supplement: Figure S2 — PCR analysis of genomic DNA isolated from several tissues. Three primer sets were used to amplify small (a, 292-bp), middle (b, 1509-bp), and large (c, 3158-bp) fragments for PCR. See Figure S4 for further details. Genomic DNA (T: tail, B: brain, H: heart, L: liver) was used as a template for PCR in zinc-finger nuclease-modified founders (numbers 28, 35, 40, and 53) and G1 rats. PCR fragments were electrophoresed through a 1–4% agarose gel. M: DNA molecular weight marker φX174-HaeIII digest or Lambda DNA-HindIII digest. (6.28 MB TIF) [file pone.0008870.s002.tif]

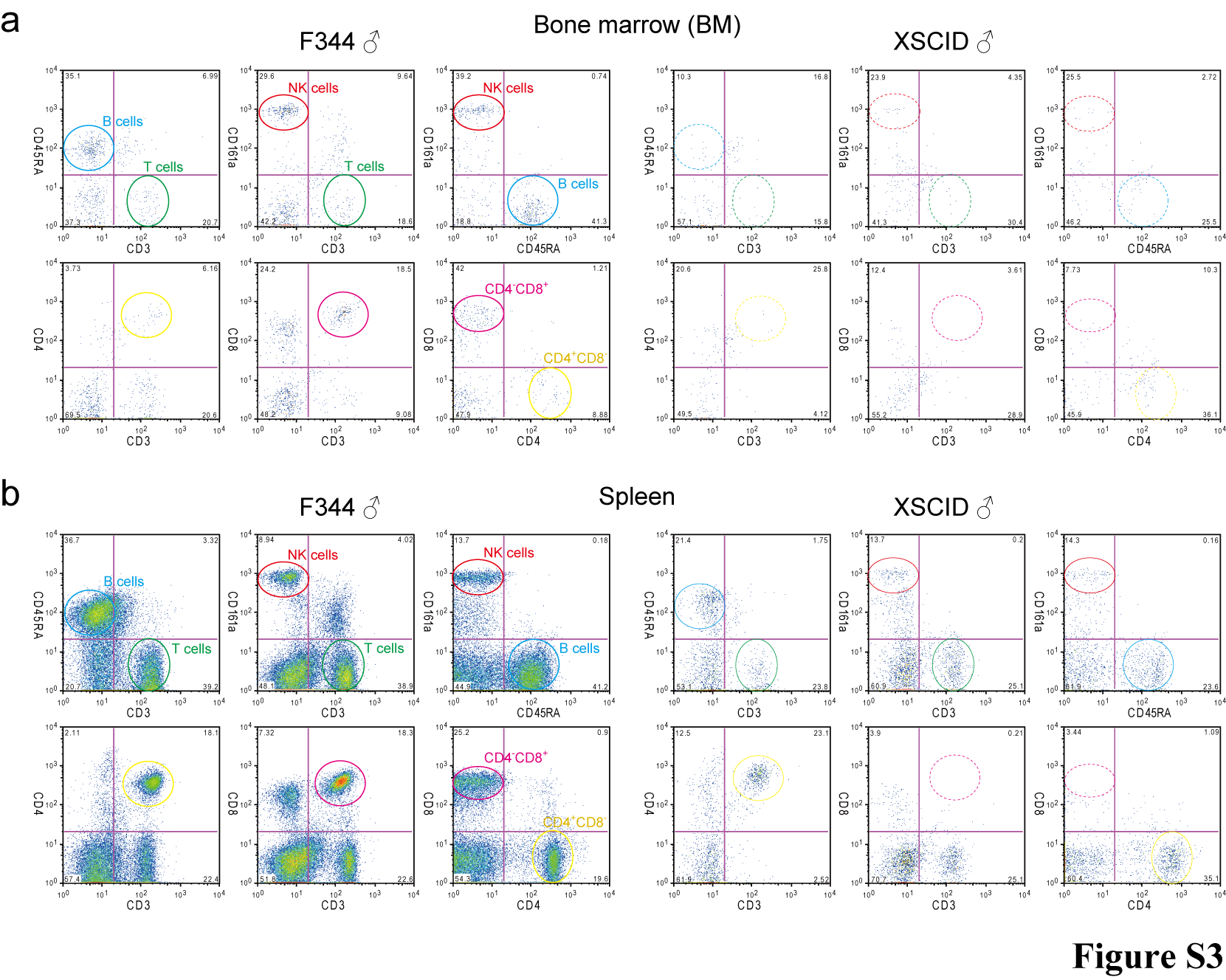

Supplement: Figure S3 — Flow cytometric analysis of bone marrow lymphocyte cells (a) and spleen lymphocyte cells (b) from five-week-old F344 and X-SCID rats. Dot plots represent CD3, CD45RA, and CD161a for discrimination of T-, B-, and NK cell subpopulations; and CD3, CD4, and CD8 for demarcation of T cell subpopulations. The numbers shown in quadrants are mean percentages. Circled areas indicate cell populations referred to in the text. (6.66 MB TIF) [file pone.0008870.s003.tif]

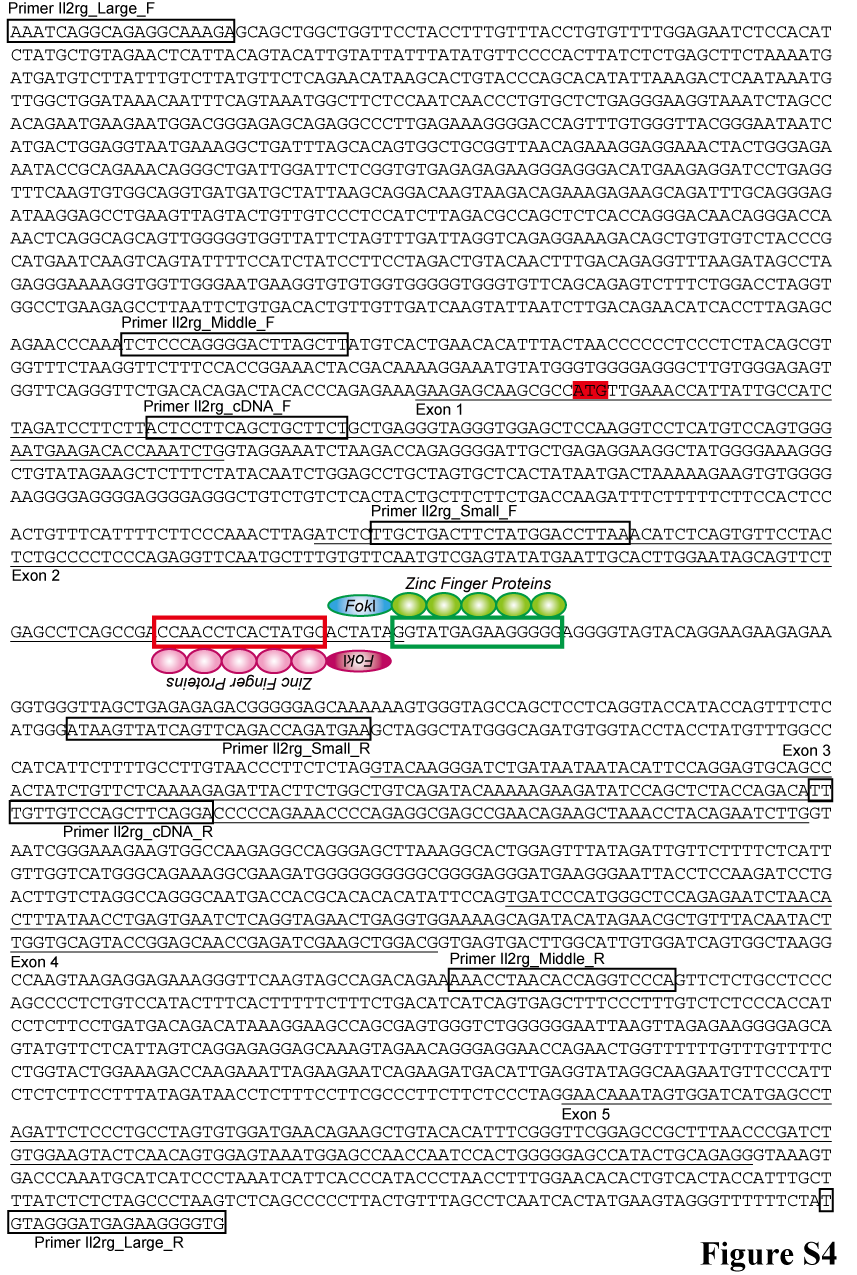

Supplement: Figure S4 — Zinc-finger nuclease pairs designed against the Il2rg locus and primer sequences used for PCR analysis for the Il2rg gene. Each exon is underlined. The start codon is indicated by a red box. The three primer sets (small, middle, and large) used for the PCR analysis of Il2rg are shown by boxes. Primers used for the RT-PCR are shown as cDNA. (3.32 MB TIF) [file pone.0008870.s004.tif]
