# Supplementary material for: Generation of Knockout Rats with X-Linked Severe Combined Immunodeficiency (X-SCID) Using Zinc-Finger Nucleases
Source: PLoS One. 2010 Jan 25;5(1):e8870. doi: 10.1371/journal.pone.0008870 (PMC2810328; doi:10.1371/journal.pone.0008870)
Supplement: Table S1 — Potential zinc-finger nuclease off-target sites. (0.14 MB DOC) [file pone.0008870.s005.doc]

| **Table S1. Potential ZFN off-target sites** | | | |  |  |  |
| --- | --- | --- | --- | --- | --- | --- |
| ID | Chr No. | Start Pos. | Sequence^a^ | No. Of Mismatch | Homodimer (+)/ Heterodimer (-) | Gene |
| 1 | chr6 | 137235447 | CACAACCTCCCTATGCCCAGAGCAGAGCAGGATGCT | 6 | + | - |
| 2 | chr18 | 48311482 | ACCAGCATCTCTATGAAGGAAACCTATGAGGTTGGA | 7 | + | - |
| 3 | chr6 | 74455695 | CCCCCACCTCTTACCTGGTGGCATAGGAGGGCTGA | 7 | - | *Egln3* |
| 4 | chr8 | 45215130 | CTCCCAGCTCAGCCCTTGATGCATAGCAGGTTGGC | 7 | - | - |
| 5 | chr11 | 9014652 | CCTACTTCTCATTCCTTAGAGCAATAGAGGTTGGT | 6 | - | - |
| 6 | chr5 | 107626344 | ACAAACTTCTCTCTGTCTCGTGTATAGGGAGGTGGGT | 6 | + | *RGD1311849_predicted* |
| 7 | chrX | 135085355 | CCGAACCTCCCTCTGCCAATAGGCATAGATGAGTGGGT | 7 | + | *Gpr119* |
| 8 | chr1 | 143289477 | accatcctccactgttggtgggattggagattggt | 7 | + | - |
| 9 | chr3 | 30383515 | ACCAAGCTTCTCTCTGCAAAGCATAGTAGGTTGTT | 7 | + | - |
| 10 | chr14 | 6221169 | AGCAAcctccattggtggtgggattggaagttggt | 7 | + | - |
| 11 | chr2 | 176120761 | TCCGACTTCCTCTGCCTAGCCGCATAGAGAGGTCAGT | 6 | + | - |
| 12 | chr20 | 21671641 | CCCAGCCCCCTATGCACAGGGGCACAACTAGGTTAGT | 7 | + | - |
| 13 | chr5 | 65817685 | ACCAAGCTCCCAGTGCTTGACAGAATAGGAGCTTGGC | 6 | + | - |
| 14 | chr16 | 9552000 | ACCCACCTCCCTAAGCAAGTAGGCCTCTGACTTTGGT | 7 | + | - |
| 15 | chr7 | 97927861 | GCCCCTACTCAGTCCATCCATCCATGGATAGGTTGGT | 7 | - | - |
| 16 | chr8 | 60327255 | CCCCCTCCTCAGCCCCTCCTAGCATAGCTAGATAGTT | 7 | - | - |
| ^a^Bases differing from the consensus target sequence are shown in red. *Fok*I catalytic sequences are shown in green. | | | | | | |
