# Supplementary material for: Generation of Knockout Rats with X-Linked Severe Combined Immunodeficiency (X-SCID) Using Zinc-Finger Nucleases
Source: PLoS One. 2010 Jan 25;5(1):e8870. doi: 10.1371/journal.pone.0008870 (PMC2810328; doi:10.1371/journal.pone.0008870)
Supplement: Table S2 — Backcrossing of zinc-finger nuclease-modified founders to F344/Stm rats. (0.16 MB DOC) [file pone.0008870.s006.doc]

| **Table S2. Backcrossing of ZFN-modified founders to F344/Stm rats** | | | | | | | |
| --- | --- | --- | --- | --- | --- | --- | --- |
| Mother | Genotypes | Father | Genotypes | Offspring | Genotypes | | Predicted genotypes of founders^a^ |
| No27 | ∆3 | F344 | + / Y | 4 (♂1, ♀3) | ♂ | 1( ∆3 / Y ) | monoallelic homozygous |
|  |  |  |  |  | ♀ | 3 ( ∆3 / + ) |
| No38 | ∆4, ∆5 | F344 | + / Y | 10 (♂5, ♀5) | ♂ | 3 ( ∆4 / Y ), 2 ( ∆5 / Y ) | biallelic heterozygous or germline chimera |
|  |  |  |  |  | ♀ | 3 ( ∆4 / + ), 2 ( ∆5 / + ) |
| No47 | ∆3, ∆660 | F344 | + / Y | 9 (♂3, ♀6) | ♂ | 1 ( ∆3 / Y ), 2 ( ∆660 / Y ) | biallelic heterozygous or germline chimera |
|  |  |  |  |  | ♀ | 1 ( ∆3 / + ), 5 ( ∆660 / + ) |
| No56 | ∆332 | F344 | + / Y | 3 (♂1, ♀2) | ♂ | 1 ( ∆332 / Y ) | monoallelic homozygous |
|  |  |  |  |  | ♀ | 2 ( ∆332 / + ) |
| No74 | ∆66, ∆1097 | F344 | + / Y | 12 (♂8, ♀4) | ♂ | 3 ( ∆66 / Y ), 5 ( ∆1097 / Y ) | biallelic heterozygous or germline chimera |
|  |  |  |  |  | ♀ | 1 ( ∆66 / + ), 3 ( ∆1097 / + ) |
| F344 | + / + | No33 | ∆39, ∆487 / Y | 12 (♂3, ♀9) | ♂ | 3 ( + / Y ) | somatic chimera |
|  |  |  |  |  | ♀ | 9 ( ∆487 / + ) |
| F344 | + / + | No35 | ∆4, ∆381 / Y | 13 (♂7, ♀6) | ♂ | 7 ( + / Y ) | somatic chimera |
|  |  |  |  |  | ♀ | 6 ( ∆381 / + ) |
| F344 | + / + | No53 | ∆13, ∆705 / Y | 9 (♂4, ♀5) | ♂ | 4 ( + / Y ) | germline chimera |
|  |  |  |  |  | ♀ | 1 ( ∆13 / + ), 4 ( ∆705 / + ) |
| ^a^The genotypes of ZFN-modified founders were infered from the genotypes of their offspring. | | | | | | | |
