# Supplementary material for: Generation of Knockout Rats with X-Linked Severe Combined Immunodeficiency (X-SCID) Using Zinc-Finger Nucleases
Source: PLoS One. 2010 Jan 25;5(1):e8870. doi: 10.1371/journal.pone.0008870 (PMC2810328; doi:10.1371/journal.pone.0008870)
Supplement: Table S3 — Intercrossing of zinc-finger nuclease-modified founders between males and females. (0.08 MB DOC) [file pone.0008870.s007.doc]

| **Table S3. Intercrossing of ZFN-modified founders between males and females** | | | | | | |
| --- | --- | --- | --- | --- | --- | --- |
| Mother | Genotypes | Father | Genotypes | Offspring | Genotypes | |
| No28 | ∆13, ∆488 | No53 | ∆13, ∆705 /Y | 10 (♂5, ♀5) | ♂ | 2 ( ∆13 / Y ), 3 ( ∆488 / Y ) |
|  |  |  |  |  | ♀ | 2 ( ∆13 / ∆705 ), 1 ( ∆488 / ∆13 ), 2 ( ∆488 / ∆705 ) |
| No40 | ∆4, ∆789, ∆1067 | No35 | ∆4, ∆381 /Y | 9 (♂2, ♀7) | ♂ | 2 ( ∆4 / Y ) |
|  |  |  |  |  | ♀ | 5 ( ∆4 / ∆381 ), 2 ( ∆1067 / ∆381 ) |
