# Supplementary material for: Generation of Knockout Rats with X-Linked Severe Combined Immunodeficiency (X-SCID) Using Zinc-Finger Nucleases
Source: PLoS One. 2010 Jan 25;5(1):e8870. doi: 10.1371/journal.pone.0008870 (PMC2810328; doi:10.1371/journal.pone.0008870)
Supplement: Table S4 — Peripheral blood profiles of Il2rg-deficient (X-SCID) rats. (0.09 MB DOC) [file pone.0008870.s008.doc]

| **Table S4. Peripheral blood (PB) profiles of *Il2rg*-deficient (X-SCID) rats** | | | | | | | | | |
| --- | --- | --- | --- | --- | --- | --- | --- | --- | --- |
|  | WBC | RBC | Hｂ | Hｔ | MCV | MCH | MCHC | Platelet | Reti. |
| Strain | （×10^3^/µL） | （×10^6^/µL） | （g/dL） | （%） | （ｆL） | （pg） | （g/dL） | （×10^3^/µL） | (%) |
| XSCID (n=5) | 2.4 ± 1.1 | 6.6 ± 0.4 | 12.4 ± 0.6 | 38.6 ± 2.2 | 58.9 ± 0.9 | 19.0 ± 0.3 | 32.1 ± 0.2 | 844.8 ± 212.4 | 8.4 ± 1.2 |
| F344/Stm (n=6) | 3.2 ± 0.5 | 6.0 ± 0.5 | 11.7 ± 0.8 | 36.5 ± 1.7 | 61.4 ± 2.0 | 19.6 ± 0.2 | 31.9 ± 0.7 | 856.3 ± 305.9 | 10.5 ± 2.0 |
| There was no statistically significant difference between the two strain for any parameters (t-test). WBC: white blood cells, RBC: red blood cells, Hb: hemoglobin, Ht: hematocrit, MCV: mean corpuscular volume, MCH: mean corpuscular hemoglobin, MCHC: mean corpuscular hemoglobin concentration, Reti.: reticulocyte ratio. | | | | | | | | | |
