# Supplementary material for: Generation of Knockout Rats with X-Linked Severe Combined Immunodeficiency (X-SCID) Using Zinc-Finger Nucleases
Source: PLoS One. 2010 Jan 25;5(1):e8870. doi: 10.1371/journal.pone.0008870 (PMC2810328; doi:10.1371/journal.pone.0008870)
Supplement: Table S5 — Differential counts of the white blood cells of Il2rg-deficient (X-SCID) rats. (0.07 MB DOC) [file pone.0008870.s009.doc]

| **Table S5. Differential counts of the white blood cells (WBC) of *Il2rg*-deficient (X-SCID) rats** | | | | | | |
| --- | --- | --- | --- | --- | --- | --- |
|  | Bas. | Eos. | Neut | Lym. | Mon. | Others |
| Strain | (%) | (%) | (%) | (%) | (%) | (%) |
| XSCID (n=5) | 0.3 ± 0.2 | 1.9 ± 1.1 | **57.0 ± 7.2 | **30.7 ± 8.1 | *5.8 ± 1.0 | 4.6 ± 2.4 |
| F344/Stm (n=6) | 0.3 ± 0.2 | 1.3 ± 0.7 | 18.6 ± 4.5 | 76.5 ± 4.8 | 2.3 ± 0.3 | 1.1 ± 0.3 |
| *P < 0.01 and **P < 0.001 indicated in comparison with control F344 (t-test). | | | | | | |
