# Supplementary material for: Generation of Knockout Rats with X-Linked Severe Combined Immunodeficiency (X-SCID) Using Zinc-Finger Nucleases
Source: PLoS One. 2010 Jan 25;5(1):e8870. doi: 10.1371/journal.pone.0008870 (PMC2810328; doi:10.1371/journal.pone.0008870)
Supplement: Table S6 — Primer sequences for zinc-finger nuclease off-target analysis. (0.14 MB DOC) [file pone.0008870.s010.doc]

| **Table S6. Primer sequences for ZFN off-target analysis** | | |  |
| --- | --- | --- | --- |
| No. | Name | Sequence | PCR size |
| 1 | Il2rg_OffT_K1F | AGGCCCCTCATAAACGAGAT | 376 |
|  | Il2rg_OffT_K1R | GGTCCCTCTGGAACCCTTAG |  |
| 2 | Il2rg_OffT_K2F | CATCCCAGCATTCTTTGCTT | 359 |
|  | Il2rg_OffT_K2R | CTGTCAGTGTTCCGTCAGGA |  |
| 3 | Il2rg_OffT_K3F | GGCTCCTTCAATTTCCATCA | 324 |
|  | Il2rg_OffT_K3R | CCTCCTGTGAGACCAGGAAA |  |
| 4 | Il2rg_OffT_K4F | GTTGCACCGTCTCATTCCTT | 368 |
|  | Il2rg_OffT_K4R | TGTGTTGCAGCTTTGTCCTC |  |
| 5 | Il2rg_OffT_K5F | AATGTCGAATGCCTGTCCTT | 267 |
|  | Il2rg_OffT_K5R | ATGCGAGACTGGACCAAGTT |  |
| 6 | Il2rg_OffT_K6F | CCCTATCAAAAGGCACCTGA | 250 |
|  | Il2rg_OffT_K6R | ACACATCTTCCCAGGGTCTG |  |
| 7 | Il2rg_OffT_K7F | GGCACTTGGAAACATCCATT | 285 |
|  | Il2rg_OffT_K7R | CACAAATGCTGCCTCTACCA |  |
| 8 | Il2rg_OffT_K8F | ATGGGGTTCGGAGCTAAACT | 396 |
|  | Il2rg_OffT_K8R | CCCTTCAACAGAGGAATGGA |  |
| 9 | Il2rg_OffT_K9F | CAAAGGCTCCACAGAGAAGG | 312 |
|  | Il2rg_OffT_K9R | AGTTATTTCATCCCGCATCG |  |
| 10 | Il2rg_OffT_K10F | TGCATGTGACAAAATTGCCTA | 308 |
|  | Il2rg_OffT_K10R | GTATATGCCCAGGGGTGATG |  |
| 11 | Il2rg_OffT_K11F | TGCTACATGAGACCCCATCA | 333 |
|  | Il2rg_OffT_K11R | GACCTGGAATCTGGAACCAA |  |
| 12 | Il2rg_OffT_K12F | TCCTCTGGCCCCACATATAG | 301 |
|  | Il2rg_OffT_K12R | GAAGCGGAGAAGGGAGAATC |  |
| 13 | Il2rg_OffT_K13F | AGATGGAGTTTGCTGCTTCC | 302 |
|  | Il2rg_OffT_K13R | CAGTGTTCAACCTGCCTAGC |  |
| 14 | Il2rg_OffT_K14F | GAGGTAACATGACCCCATGC | 373 |
|  | Il2rg_OffT_K14R | AAGCTCCAGTGACCCCCTAT |  |
| 15 | Il2rg_OffT_K15F | CAAACCCCTGCTAAAATGGA | 288 |
|  | Il2rg_OffT_K15R | CTGAGGCTTTGTCTCCCTGT |  |
| 16 | Il2rg_OffT_K16F | AGCCATTATGCTCACCCAAG | 258 |
|  | Il2rg_OffT_K16R | CAATGAGAGCACTCCAAGCA |  |
